# Cooperative miRNA-dependent PTEN regulation drives resistance to BTK inhibition in B-cell lymphoid malignancies

**DOI:** 10.1038/s41419-021-04353-9

**Published:** 2021-11-08

**Authors:** Isha Kapoor, Juraj Bodo, Brian T. Hill, Alexandru Almasan

**Affiliations:** 1 Department of Cancer Biology, Lerner Research Institute, Cleveland, OH USA; 2 Department of Laboratory Medicine, Institute of Pathology and Laboratory Medicine, Cleveland, OH USA; 3 Department of Hematology and Medical Oncology, Taussig Cancer Institute, Cleveland, OH USA; 4 Department of Radiation Oncology, Taussig Cancer Institute, Cleveland Clinic, Cleveland, OH 44195 USA

**Keywords:** Cancer, Chronic lymphocytic leukaemia

## Abstract

Aberrant microRNA (miR) expression plays an important role in pathogenesis of different types of cancers, including B-cell lymphoid malignancies and in the development of chemo-sensitivity or -resistance in chronic lymphocytic leukemia (CLL) as well as diffuse large B-cell lymphoma (DLBCL). Ibrutinib is a first-in class, oral, covalent Bruton’s tyrosine kinase (BTK) inhibitor (BTKi) that has shown impressive clinical activity, yet many ibrutinib-treated patients relapse or develop resistance over time. We have reported that acquired resistance to ibrutinib is associated with downregulation of tumor suppressor protein PTEN and activation of the PI3K/AKT pathway. Yet how PTEN mediates chemoresistance in B-cell malignancies is not clear. We now show that the BTKi ibrutinib and a second-generation compound, acalabrutinib downregulate miRNAs located in the 14q32 miRNA cluster region, including miR-494, miR-495, and miR-543. BTKi-resistant CLL and DLBCL cells had striking overexpression of miR-494, miR-495, miR-543, and reduced PTEN expression, indicating further regulation of the PI3K/AKT/mTOR pathway in acquired BTKi resistance. Additionally, unlike ibrutinib-sensitive CLL patient samples, those with resistance to ibrutinib treatment, demonstrated upregulation of 14q32 cluster miRNAs, including miR-494, miR-495, and miR-543 and decreased *pten* mRNA expression. Luciferase reporter gene assay showed that miR-494 directly targeted and suppressed PTEN expression by recognizing two conserved binding sites in the PTEN 3′-UTR, and subsequently activated AKT^Ser473^. Importantly, overexpression of a miR-494 mimic abrogated both PTEN mRNA and protein levels, further indicating regulation of apoptosis by PTEN/AKT/mTOR. Conversely, overexpression of a miR-494 inhibitor in BTKi-resistant cells restored PTEN mRNA and protein levels, thereby sensitizing cells to BTKi-induced apoptosis. Inhibition of miR-494 and miR-495 sensitized cells by cooperative targeting of *pten*, with additional miRNAs in the 14q32 cluster that target *pten* able to contribute to its regulation. Therefore, targeting 14q32 cluster miRNAs may have therapeutic value in acquired BTK-resistant patients via regulation of the PTEN/AKT/mTOR signaling axis.

## Introduction

B-cell lymphoid malignancies, including chronic lymphocytic leukemia (CLL) and diffuse large B-cell lymphoma (DLBCL), the most prevalent subtypes of non-Hodgkin lymphoma (NHL), are characterized by chronic activation of the B-cell receptor (BCR) signaling [1, 2]. Bruton’s tyrosine kinase (BTK) is a central kinase in the BCR axis that drives a signaling cascade leading to activation of NF-κB and phosphatidylinositol-3-kinase (PI3K) pro-survival pathways in CLL and the activated B-cell (ABC) subset of DLBCL [1, 3].

Ibrutinib, an FDA-approved, first-in-class orally administered BTK inhibitor that binds covalently to the C481 residue of BTK, has demonstrated impressive clinical activity in newly diagnosed and treatment-relapsed/refractory patients with CLL and many subtypes of NHL [4, 5]. However, ibrutinib also binds to other homologous cysteine-containing kinases, such as ITK, EGFR, TEC, and BMX, which result in toxic off-target side-effects, eventually leading to discontinuation of ibrutinib [6–8]. The clinical activity of ibrutinib as a single agent in DLBCL has a preferential benefit for patients with ABC-DLBCL but its utility is limited [9–12]. Despite the efficacy of ibrutinib, clinical responses are variable/partial, often leading to drug resistance and aggressive relapse of the disease. Up to 5% of ibrutinib-treated patients progress with more aggressive ABC-DLBCL [9, 10]. Toxicities, such as atrial fibrillation, bleeding, or arterial hypertension, albeit limited, caused by inhibition of other non-BTK targets, such as ITK and EGFR underscores the need for more selective BTK inhibitors with fewer off-target effects [6, 11].

Unlike ibrutinib, acalabrutinib, is an FDA-approved second-generation, highly selective, potent, covalent BTK inhibitor with minimal off-target effects [6]. Interestingly, these BTK inhibitors showed a similar preclinical activity profile, molecular, and biologic effects in primary CLL cells [6, 13–15]. Acalabrutinib monotherapy has shown good tolerability and efficacy in treatment-naïve, relapsed/refractory CLL patients. As a single agent, acalabrutinib demonstrated a high response rate (~81%) in ibrutinib-intolerant CLL patients [8]. Recently presented head-to-head comparison shows similar clinical activity but improved safety profile of acalabrutinib vs ibrutinib [16]. Recent studies showed constitutive activation of the PI3K/AKT pathway in 25–52% of DLBCL patients, and correlated overexpression of phosphorylated Akt (pAKT) with significantly poorer progression-free survival in approximately one-fourth of DLBCL patients [17]. We and others have previously shown that downregulation of PTEN, a major negative regulator of the PI3K/AKT signaling is significantly associated with chemotherapy resistance and poor survival in patients with DLBCL with AKT hyperactivation [17–19]. Yet, how PTEN mediates resistance to BTK inhibition in B-cell malignancies is not clear.

Following prolonged treatment, CLL and DLBCL patients can acquire resistance to BTK inhibitors, ibrutinib or acalabrutinib, through mutations in BTK and its substrate phospholipase C gamma 2 (PLCG2), MYD88, and CARD11 [18, 20]. In addition to the acquisition of these mutations, other mechanisms can confer resistance to BTK inhibition, such as upregulation of druggable survival pathways, clonal evolution due to other genetic alterations [18], or aberrant expression of miRNAs [21, 22]. Such mechanisms may be overcome by rational therapeutic combinations of targeted agents that block adaptive pathways promoting drug resistance. Several studies have reported the involvement of aberrant expression of micro-RNAs (miRNAs) in the development of chemo-sensitivity or -resistance in various cancers, including CLL and DLBCL [21, 23]. miRNAs are small (~20–22 nucleotides) noncoding regulatory RNAs that bind to a specific target mRNA through a sequence that is complementary to the 3′-UTR of the target mRNA [23]. Several miRNAs regulate oncogenic or tumor-suppressive pathways, such as the NF-*κ*B or BCR signaling cascade in B-cell malignancies [21, 22, 24, 25]. Our previous studies have shown aberrant regulation of miR-377 in germinal center-type DLBCL that targets BCL-xL, and thus drives acquired resistance to BCL-xL inhibition by venetoclax [23].

Therefore, we investigated the underlying molecular signatures of BTKi resistance in sensitive vs acquired acalabrutinib-resistant (Aca-R) and ibrutinib-resistant (IB-R) cells following chronic exposure to these therapeutics. By comparing sensitive vs acquired BTKi-R cells, we have defined BTKi-R as a 14q32 miRNA cluster-dependent regulation of PTEN/AKT/mTOR in CLL and DLBCL in the absence of BTK or PLCG2 mutations. Our data reveal novel mechanistic insights into the role of cooperative PTEN-targeting by 14q32 cluster miRNAs: miRNA-494 and miR-495, as well as miR-453, miR-899, miR-737, and miR-433 in BTKi-R cells. These findings provide a rationale for cooperative inhibition of overexpressed oncogenic miRNAs to overcome resistance to BTK inhibition in lymphoid malignancies by upregulation of PTEN leading to AKT/mTOR activation.

## Results

### Acquired resistance to chronic BTK inhibition leads to upregulation of 14q32 cluster miRNAs

**Fig. 1:**
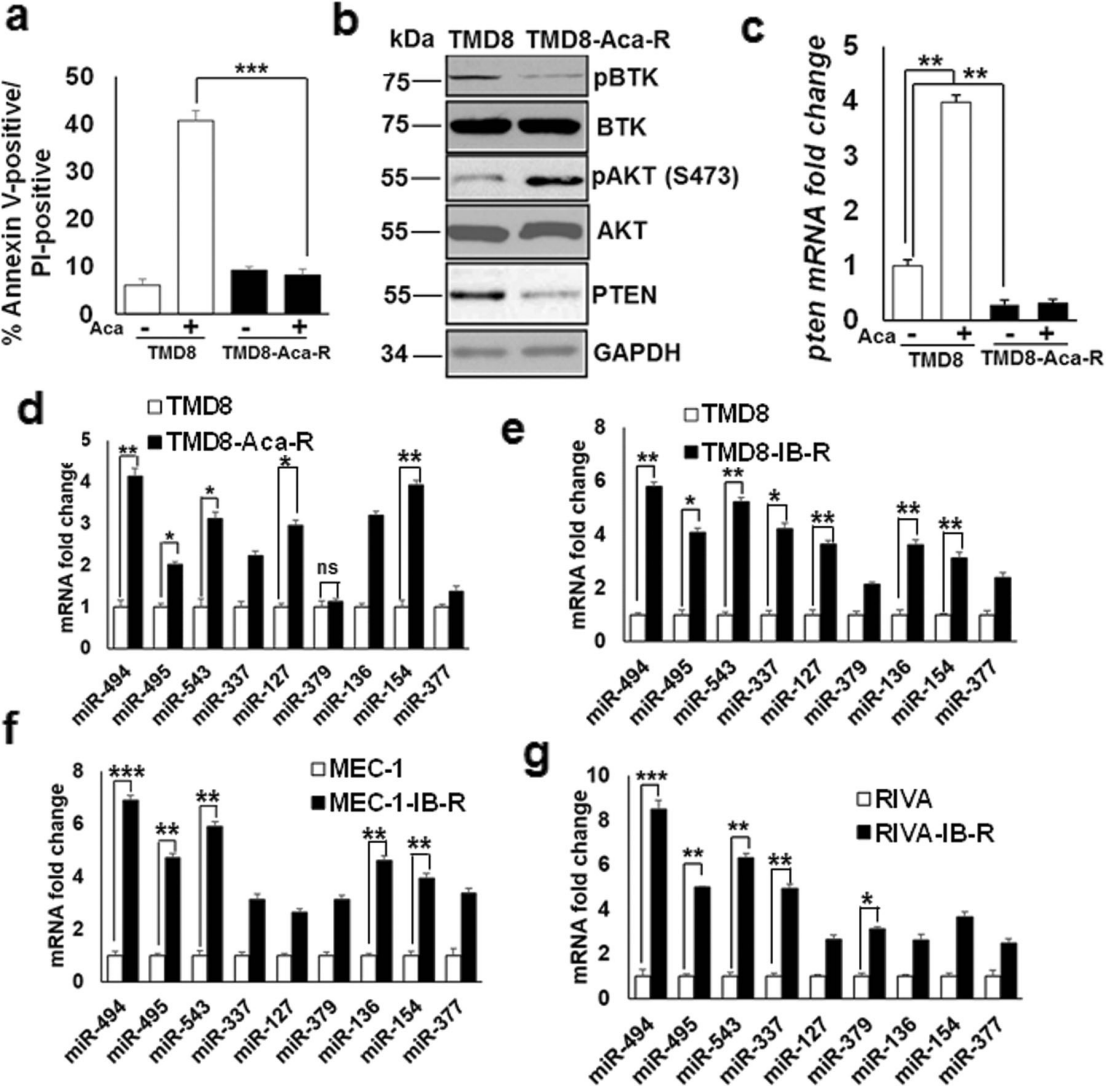
BTK inhibition downregulates miRNAs in the 14q32 cluster region in CLL and DLBCL cells. **a** Cell death analysis in parental TMD8 and acalabrutinib-resistant derivatives (TMD8-Aca-R) in response to 24 h acalabrutinib treatment determined by Annexin V/PI staining. All data were expressed as mean ± SD of the percentage of cell death. **b** Expression levels of pAKT^Ser473^, AKT, and PTEN in whole-cell extracts of untreated parental and Aca-R TMD8 cells. GAPDH was used as a loading control. **c** mRNA fold change of *pten* in parental vs Aca-R TMD8 cells with or without acalabrutinib (5 µM) for 48 h. **d** Relative expression changes of miRNAs in the 14q32 domain cluster, as determined by qRT-PCR in untreated parental and Aca-R TMD8 cells. miRNAs fold change in Aca-R cells is normalized to untreated parental cells. SD is indicated as error bars (*N* = 3). **e**–**g** miRNAs fold change in parental vs IB-R TMD8, MEC-1, and RIVA cells. miRNAs fold change in ibrutinib-treated cells is normalized to untreated parental cells. (*p < 0.05, **p < 0.01, ***p < 0.001). Standard deviation (SD) is indicated as error bars (*N* = 3).

Aca-R ABC-DLBCL (TMD8), IB-R ABC-DLBCL (RIVA, TMD8), and CLL (MEC-1) cell lines were generated by culturing the parental cell lines in vitro with progressively increasing concentrations of acalabrutinib or ibrutinib, as previously described [18]. Cell viability analysis showed a ~40% increase in cell death in TMD8 (Fig. 1a), but not in Aca-R-derivative cells after 24 h of acalabrutinib treatment. Similarly, MTS assays showed a high sensitivity to increasing concentrations of acalabrutinib administered for 72 h with an IC_50_ of 78 nM for TMD8 cells. These Aca-R-derivative cells were resistant to much higher concentrations than the IC_50_ of the parental cells (Supplementary Fig. S1a).

To investigate the mechanism of Aca-R, we examined the expression pattern of PTEN in parental and resistant cells. Immunoblot analyses show that the levels of PTEN were low in resistance (TMD8-Aca-R) compared to parental cells (Fig. 1b). Additionally, levels of pAKT (AKT^Ser473^) were upregulated in TMD8-Aca-R compared to parental cells. Importantly, pBTK (BTK^Y223^) levels were diminished, indicating that chronic acalabrutinib treatment blocks BTK activation in Aca-R cells. Notably, the qRT-PCR analysis indicated that *pten* mRNA levels were decreased by ~4-fold, even after acute treatment with acalabrutinib in TMD8-Aca-R vs parental cells, indicating that the reduced PTEN levels could be attributed to decreased *pten* mRNA levels (Fig. 1c). Taken together, these findings indicate the importance of the PTEN/AKT axis in mediating Aca-R.

We have previously shown that acquired resistance to ibrutinib is associated with PTEN downregulation and activation of the PI3K/AKT pathway [18]. To investigate the mechanism of resistance to BTK inhibition, we examined the expression patterns of miRNAs located in the 14q32 cluster that we previously found to be involved in the resistance to BCL-xL inhibition in CLL and DLBCL [23]. Examination of the expression patterns of nine miRNAs located in the 14q32 cluster by qRT-PCR analyses indicated their increased expression in both Aca-R (Fig. 1d) and IB-R (Fig. 1e–g) DLBCL and CLL cell lines. Of these miRNAs, expression of miR-494 and miR-543 was increased by ~3-fold and 2-fold, respectively in TMD8-Aca-R cells (Fig. 1d). Similarly, expression of miR-494, miR-495, and miR-543 were increased by ~4.8-fold, ~3-fold, and ~2-fold, respectively in TMD-IB-R (Fig. 1e), ~6-fold, 4-fold, and 5-fold, respectively in MEC-1-IB-R (Fig. 1f) and ~7.5-fold, 4-fold, and 5-fold, respectively in RIVA-IB-R cells (Fig. 1g). Taken together, these findings indicate an association of aberrant expression of 14q32 cluster miRNAs with resistance to BTK inhibition.

### BTK inhibition downregulates 14q32 cluster miRNAs and upregulates PTEN expression

**Fig. 2:**
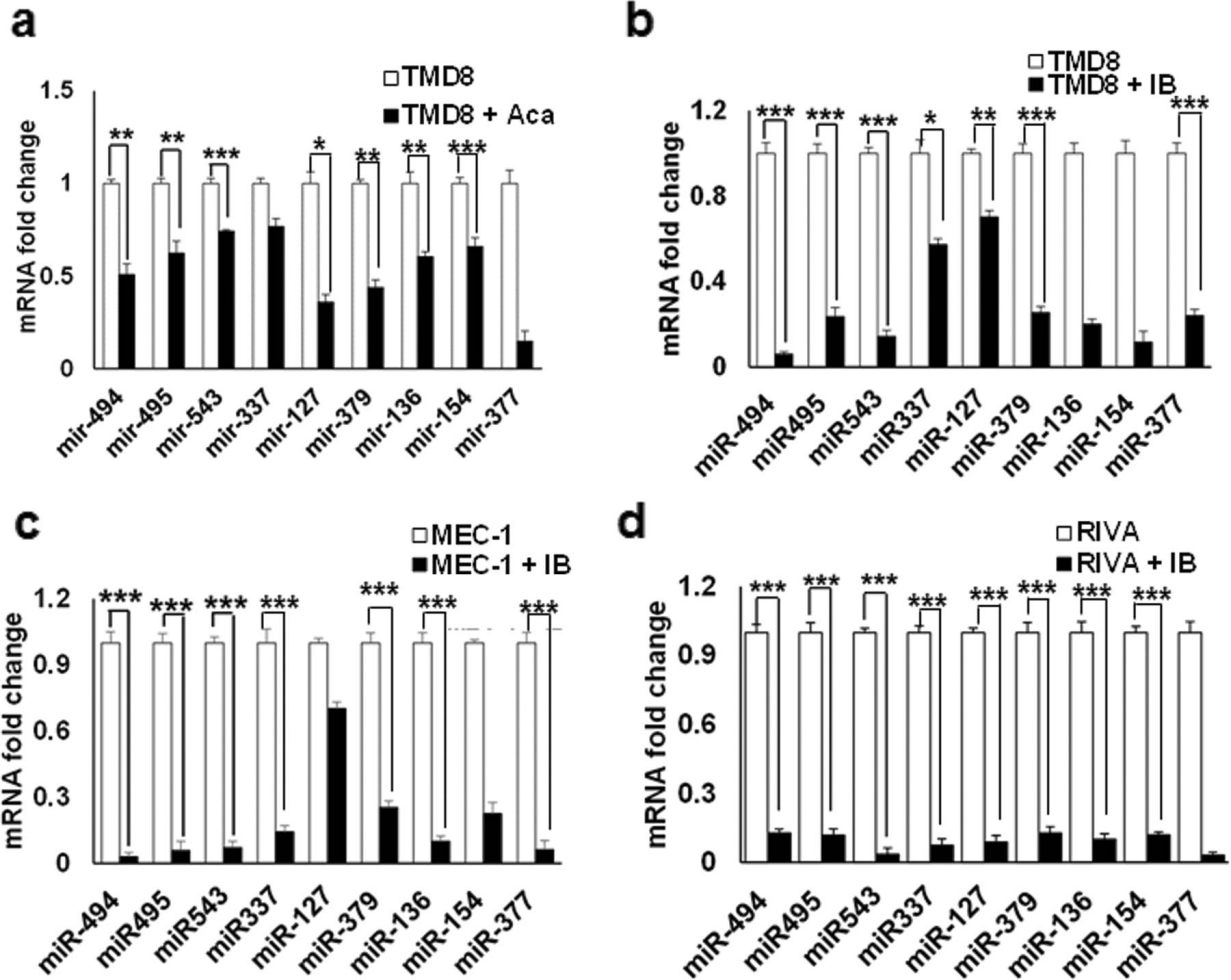
Expression of 14q32 cluster miRNAs is decreased after BTK inhibition in CLL and DLBCL. **a** Expression of nine miRNAs in the 14q32 cluster, as determined by qRT-PCR in TMD8 cells with or without acalabrutinib (5 µM) for 48 h. miRNA fold change in acalabrutinib treated cells is normalized to untreated cells. **b**–**d** Relative expression changes of nine miRNAs in the 14q32 cluster, as determined by qRT-PCR in TMD8, MEC-1, and RIVA cells treated with ibrutinib (10 µM) for 48 h. miRNAs fold change in ibrutinib-treated cells is normalized to untreated cells. (*p < 0.05, **p < 0.01, ***p < 0.001). SD is indicated as error bars (*N* = 3).

Since BTKi resistance following chronic exposure to acalabrutinib or ibrutinib resulted in increased expression of 14q32 cluster miRNAs and lower levels of PTEN, we examined the effects of acute acalabrutinib and ibrutinib treatment on 14q32 cluster miRNAs in BTKi-sensitive ABC-DLBCL and CLL cells. Parental TMD8 cells treated with acalabrutinib demonstrated downregulation of 14q32 cluster miRNAs, with decreased levels of miR-494 (~50%), miR-495 (~40%), and miR-543 (~30%) (Fig. 2a). BTK inhibition by ibrutinib in TMD8 cells resulted in ~90% reduced expression of miR-494 and miR-543, and ~80% decreased expression of miR-495 (Fig. 2b). Similarly, ~90% reduced expression of miR-494, miR-495, and miR-543 were observed in both MEC-1 (Fig. 2c) and RIVA cells (Fig. 2d) treated with ibrutinib. Taken together, these findings indicate the potential role of aberrant expression of 14q32 cluster miRNAs in mediating BTKi resistance.

### BTK inhibition decreases the expression of 14q32 cluster miRNAs and increases that of PTEN in patient-derived primary CLL cells

**Fig. 3:**
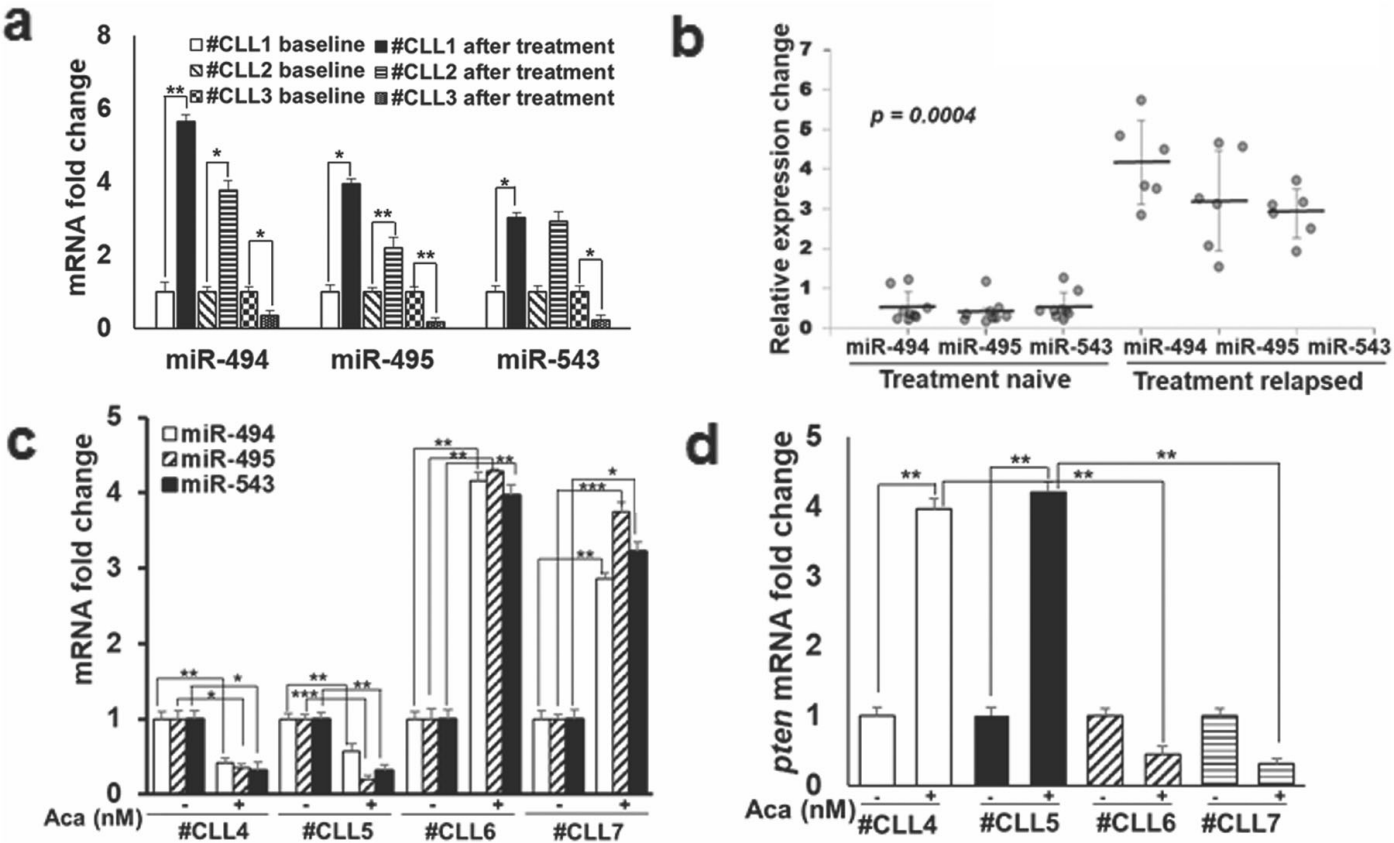
Expression of 14q32 cluster miRNAs is decreased and that of PTEN increased after BTK inhibition in patient-derived primary CLL cells. **a** Expression of miR-494, miR-495, and miR-543 was analyzed in primary cells obtained from three paired CLL patients’ samples pre- and post-ibrutinib-treated in the clinic. **b** Relative expression changes in miR-494, miR-495, and miR-543 was analyzed in primary cells obtained from nine treatment naïve vs five treatment-relapsed CLL patients’ samples after in vitro treatment with ibrutinib. Mann–Whitney nonparametric analysis was performed to compare them. Two-sided *p* value is 0.0004. **c**, **d** Relative expression of miR-494, miR-495, miR-543, and *pten* was analyzed in primary cells obtained from treatment naïve (#CLL4, #CLL5) and relapsed (#CLL6, #CLL7) CLL patients’ samples after in vitro treatment with acalabrutinib (5 µM). (*p < 0.05, **p < 0.01, ***p < 0.001). SD is indicated as error bars (*N* = 1).

Given the differences in expression of 14q32 cluster miRNAs and PTEN in Aca-R and IB-R CLL and DLBCL cells in vitro, we tested whether miRNA expression might also be altered in patient-derived primary CLL cells, in response to BTK inhibition or standard-of-care clinical therapy. qRT-PCR analysis in three paired CLL patient samples pre- and post-ibrutinib-treated in the clinic revealed a decrease in the levels of miR-494, miR-495, and miR-543 in #CLL3 (ibrutinib-sensitive) after clinical ibrutinib treatment in contrast to #CLL1 (ibrutinib-resistant) and #CLL2 (partial remission) (Fig. 3a). Additionally, in vitro ibrutinib treatment of CLL patient samples revealed an increase in the expression of miR-494, miR-495, and miR-543 in treatment-relapsed vs naïve patients (Fig. 3b). Similarly, in vitro acalabrutinib treatment of naïve (#CLL4, #CLL5) vs treatment-relapsed (#CLL6, #CLL7) CLL patients showed significant downregulation of miR-494 (58%), miR-495 (64%), and miR-543 (68%) in #CLL4 and #CLL5 (treatment naïve) (43% reduction in miR-494; 79% miR-495, and 68% miR-543) in contrast to #CLL6 and #CLL7 (treatment-relapsed) (Fig. 3c). Additionally, in vitro acalabrutinib treatment of naïve (#CLL4, #CLL5) vs treatment-relapsed (#CLL6, #CLL7) CLL patients revealed significant increase in *pten* mRNA levels in #CLL4 (2.9-fold) and #CLL5 (3.2-fold) in contrast to #CLL6 and #CLL7 (Fig. 3d). Taken together, these results indicate the role of aberrant expression of 14q32 cluster miRNAs in mediating therapeutic resistance.

### PTEN is a direct target of miR-494

**Fig. 4:**
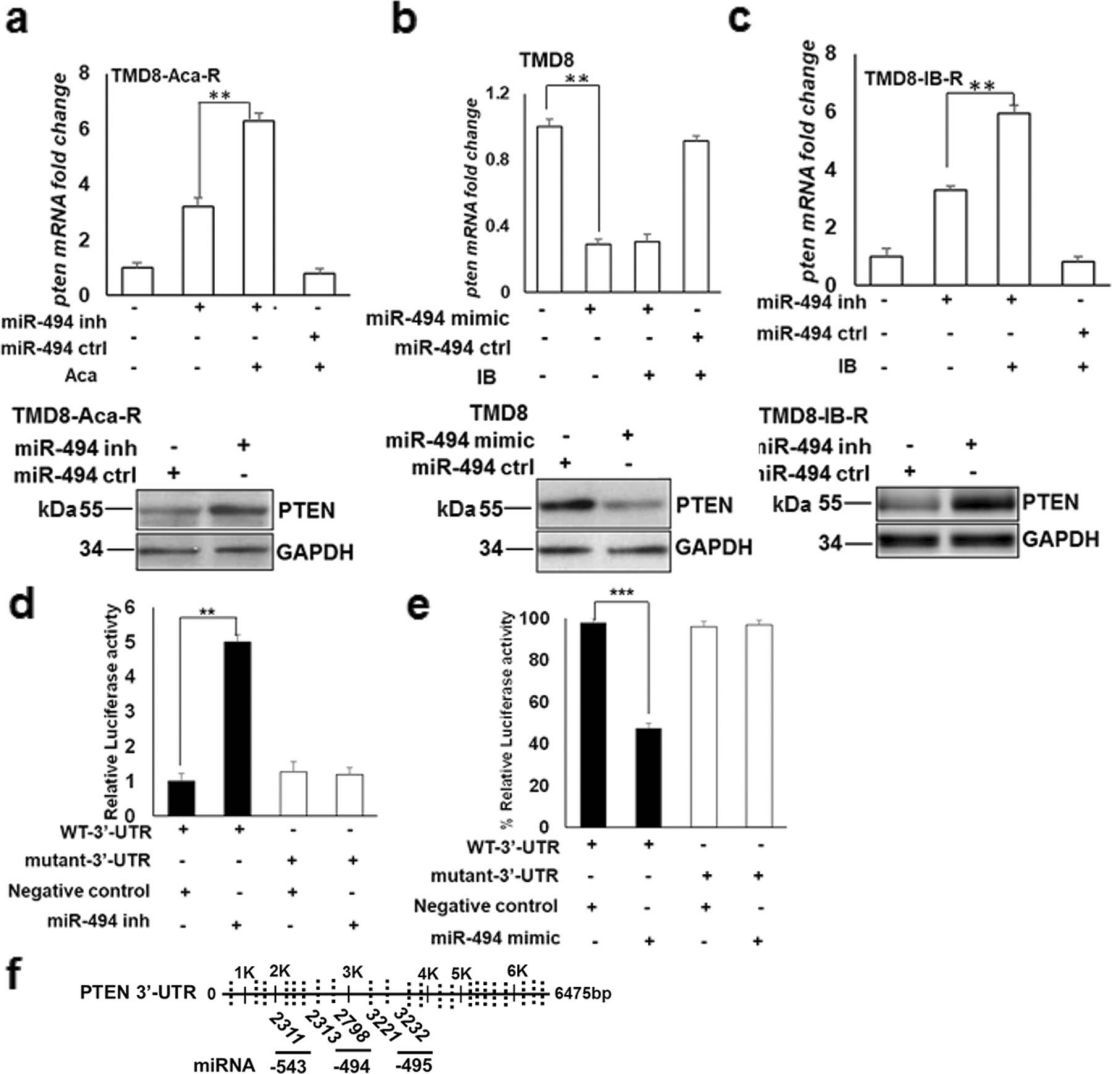
PTEN is a direct target of miR-494 in CLL and DLBCL. **a** Expression levels of PTEN mRNA (top) and protein (bottom) in TMD8-Aca-R and **b** Expression levels of PTEN mRNA (top) and protein (bottom) in TMD8 cells after transfection with miR-494 inhibitor, mimic, or negative control, as indicated. GAPDH was used as a loading control. **c** Expression levels of PTEN mRNA (top) and protein (bottom) in TMD8-IB-R cells after transfection with miR-494 inhibitor or negative control, as indicated. GAPDH was used as a loading control. **d**, **e** Luciferase reporter activity in TMD8-IB-R and TMD8 and cells co-transfected with PTEN WT-3′-UTR or mutant 3′-UTR constructs and miR-494 inhibitor or miR-494 mimic, respectively together with negative control as indicated. (**p < 0.01, ***p < 0.001). SD is indicated as error bars (*N* = 3). **f** schematic representation of the PTEN 3′-UTR with positions of targeting miRNAs. Conserved sites for miR-494, miR-495, and miR-543 are indicated in bold. Other non-conserved sites for other miRNAs, as mentioned in Table 1, are indicated as dotted lines on PTEN 3′-UTR.

Previously, we have shown that ibrutinib treatment regulates transcriptional activation of PTEN in CLL and DLBCL cells [18]. Using target prediction software to identify miRNAs that have a putative PTEN target, we found six miRNAs located in the 14q32 cluster, of which miR-494 had the highest score (Fig. 4a). Interestingly, qRT-PCR and immunoblot analysis of TMD8-Aca-R cells transfected with a miR-494 inhibitor revealed a significant increase in the expression of *pten* mRNA (upper panel) and protein (lower panel) levels (Fig. 4b). Similar results were obtained in TMD8-IB-R (Fig. 4c), MEC-1-IB-R, and RIVA-IB-R cells (Supplementary Fig. S2a, b). To confirm that miR-494 is directly involved in *PTEN* regulation, adding miR-494 mimics led to a substantial decrease in both endogenous PTEN mRNA (upper panel) and protein (lower panel) expression levels in parental TMD8 (Fig. 4d), MEC-1 and RIVA cells (Supplementary Fig. S2c, d). Additionally, the qRT-PCR analysis indicated that overexpression of miR-494 inhibitor in TMD-Aca-R, TMD8-IB-R, and MEC-1-IB-R (Supplementary Fig. S3a–c) results in increased expression of proapoptotic *bim* mRNA levels while overexpression of miR-494 mimic in MEC-1 and TMD8 cells (Supplementary Fig. S3d, e) results in decreased expression of *bim* mRNA levels. Taken together, these results indicate that both PTEN and BIM is regulated at the posttranscriptional level by miR-494 and could be a direct target.

To determine potential targets of miR-494, we performed prediction analysis of the 14q32 miRNA cluster region and *PTEN* 3′-UTR alignment using mirDB [26, 27]. This analysis identified two conserved complementary sequences at positions 2313 and 2798 in the 3′-UTR of *PTEN* mRNA with which miR-494 is likely to base-pair (Fig. 4d). To examine whether PTEN is a direct target of miR-494, luciferase reporter containing wild-type (WT) PTEN 3′-UTR or miR-494-binding site mutant PTEN 3′-UTR were transfected together with miR-494 mimic (Fig. 4e) or inhibitor (Fig. 4f) and negative control in TMD8 and TMD8-IB-R cells, respectively and luciferase activity was measured after 48 h. Ectopic miR-494 mimic expression in TMD8 cells downregulated WT-3′-UTR-associated luciferase activity by ~50% as compared with the negative control mimic (Fig. 4e). In contrast, transfection with mutant 3′-UTR luciferase reporter, miR-494 mimic expression was unable to suppress luciferase activity at all (Fig. 4d). Transfection with a miR-494 inhibitor in TMD8-IB-R cells completely reversed the luciferase activity, resulting in a 4-fold increase in wild-type WT-3′-UTR-associated luciferase activity as compared to negative control inhibitor (Fig. 4f). Taken together, these results indicate a direct binding of miR-494 to the predicted and previously reported [25] target sites in the *PTEN* 3′-UTR.

### AKT inhibition potentiates miRNA inhibition-induced apoptosis in BTK-R CLL and DLBCL

**Fig. 5:**
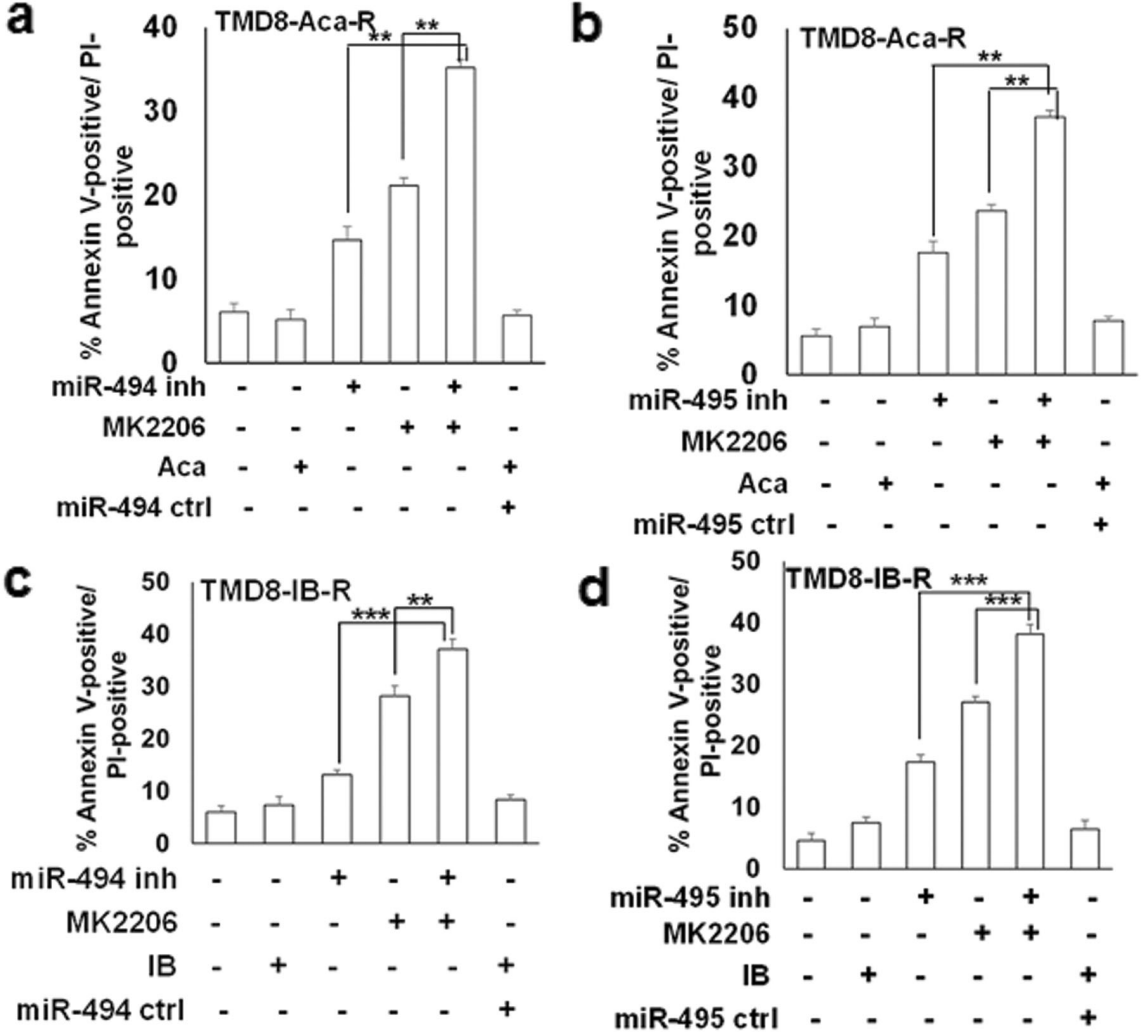
miRNA inhibition potentiates AKT-induced apoptosis in BTK inhibitor-resistant CLL and DLBCL. **a** TMD8-Aca-R cells were transfected with miR-494 (100 nM) and **b** miR-495 (50 nM) inhibitors and treated with ± MK2206 (5 µM) for 24 h. Cell death analysis was determined by Annexin V-PI staining. Control cells were treated with DMSO. TMD8-IB-R cells were transfected with miR-494 (**c**) and miR-495 (**d**) inhibitors together with miRControl and treated with MK2206 (5 µM) for 24 h. Cell viability was determined by Annexin V-PI staining. Control cells were treated with DMSO. (*p < 0.05, **p < 0.01, ***p < 0.001). All data were expressed as mean ± SD of the percentage of cell death. SD is indicated as error bars (*N* = 3).

Given that PTEN expression was downregulated by miR-494, we next examined whether these cells could be further sensitized to apoptosis by AKT inhibition, in combination with miR-494 or miR-495. Previously, we have shown that AKT activation was elevated in our IB-R cells, and PTEN was significantly downregulated, thus making IB-R cells more sensitive to induction of apoptosis by AKT inhibition [18]. Pharmacological inhibition of AKT showed ~20 and ~19% increase in apoptosis in TMD8 and Aca-R cells, respectively by inhibition of AKT together with miR-494 (Fig. 5a) or miR-495 (Fig. 5b). Similarly, inhibition of miR-494 (Fig. 5c) and miR-495 (Fig. 5d) in TMD8-IB-R cells showed ~24 and ~21% increase in apoptosis with AKT inhibition, respectively. Similar results were obtained in MEC-1-IB-R cells with inhibition of AKT in combination with miR-494 (~33%) and miR-495 (~25%) (Supplementary Fig. S4a, b) in comparison to miR-494 or miR-495 inhibition alone. Together, these results indicate the dependency of BTKi-R cells on miR-494 or miR-495-dependent regulation of PTEN/AKT and that inhibition of AKT phosphorylation/activation increases miR-494 and miR-495 inhibition-induced apoptosis in BTK-R cells.

### Cooperative miRNA inhibition potentiates apoptosis by targeting PTEN

**Fig. 6:**
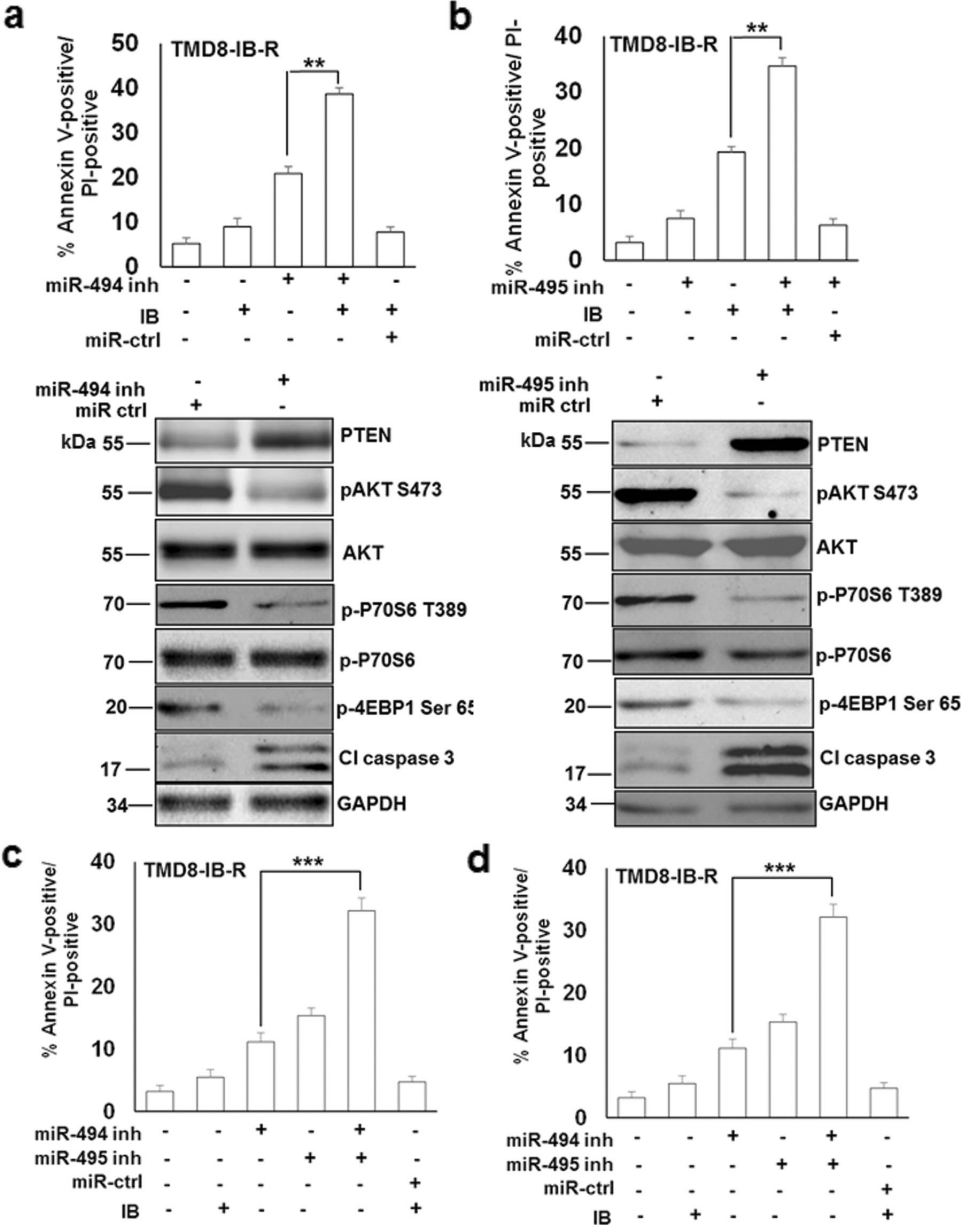
Cooperative miR-494 and miR-495 inhibition enhances cell survival through AKT/mTOR signaling. **a** TMD8-IB-R cells were transfected with miR-494 (200 nM) and **b** miR-495 (100 nM) inhibitors and treated with ± ibrutinib (10 µM) for 24 h. **(**Upper panel) Cell death analysis was determined by Annexin V-PI staining. Control cells were treated with DMSO. (**p < 0.01). SD is indicated as error bars (*N* = 3). (Lower panel) Expression levels of PTEN, pAKT Ser473, AKT, pP70S6-T389, pP70S6, p4EBP1, and cleaved caspase 3 were determined in whole-cell extracts of TMD-IB-R cells transfected with miR-494 (200 nM) and miR-495 inhibitors, respectively (100 nM) by immunoblotting. GAPDH was used as a loading control. **c** TMD8-IB-R and **d** MEC-1-IB-R cells were transfected with miR-494 (200 nM) and miR-495 (100 nM) either alone or in combination for 24 h. Cell death analysis was determined by Annexin V-PI staining. Control cells were treated with DMSO. (*p < 0.05, **p < 0.01). SD is indicated as error bars (*N* = 3).

miR-494 inhibition suppressed AKT and mTOR activity, as indicated by a decrease in pAKT^Ser473^ and inhibition of phosphorylation of downstream targets of mTORC1, p70S6^Thr389^ kinase, and p4EBP1. Notably, the expression of PTEN protein levels was increased in TMD8-IB-R cells by miR-494 inhibition. AKT and p70S6 kinase levels, however, did not change significantly (Fig. 6a). These findings indicate that an increase in PTEN protein expression in response to miR-494 inhibition in TMD8-IB-R cells was associated with decreased activity of AKT and mTOR. In addition, miR-494 inhibition resulted in increased expression of caspase 3 cleavage (Fig. 6a). Similar results were obtained in MEC-1-IB-R cells (Supplementary Fig. S5a) and miR-494 inhibition conferred a ~20% increase in ibrutinib-induced apoptosis in cells (Fig. 6a; upper panel). Similar results were obtained with miR-495 inhibition in TMD-IB-R (Fig. 6b) and MEC-l-IB-R cells (Supplementary Fig. S5b; lower panel).

Next, we examined whether these miRNAs may coordinately impact cell viability by regulating PTEN expression. Cell viability analysis in TMD8-IB-R (Fig. 6c) and MEC-1-IB-R (Fig. 6d) cells demonstrated an ~20% increase in apoptosis in cells transfected with a combination of miR-494 and miR-495 inhibitors compared to transfection with either miR-494 or miR-495 inhibitors alone, indicating a cooperative action of these miRNA inhibitors on cell survival.

## Discussion

Despite the extensive heterogeneity of B-cell lymphoid malignancies, accumulating evidence has supported the association between deregulated expression of miRNAs and therapeutic resistance in various cancers, including CLL and DLBCL [21–23, 25]. Our recent findings uniquely characterize the development of acquired IB-R by PTEN downregulation that impedes ibrutinib-induced apoptosis, as demonstrated by AKT activation [18]. In our present study, we show for the first time that similar to IB-R cells, AKT activation was elevated in our Aca-R cells and PTEN was significantly downregulated following chronic exposure to acalabrutinib. Nevertheless, how PTEN mediates resistance to BTK inhibition was unknown.

In search of the molecular mechanism responsible for decreased PTEN expression, we have uniquely characterized a novel role for 14q32 cluster miRNAs-dependent regulation of the PTEN/AKT/mTOR axis in mediating resistance to BTK inhibition in CLL and DLBCL cells in the absence of BTK or PLCG2 mutations. Interestingly, there is a growing interest in the maternally imprinted *DLK1-DIO3* region on chromosome 14q32 because ~53 miRNAs are embedded in two adjacent clusters, many of which have been reported to be deregulated in various cancers, such as, APL [28], melanoma [29], and lung adenocarcinomas [30].

Previously, we have reported miRNA-377-dependent regulation of BCL-xL in venetoclax resistance in B-cell lymphoid malignancies and germinal center-type DLBCL [23]. Here we show that chronic BTK inhibition leads to upregulation of the 14q32 miRNA cluster in CLL and DLBCL cells. Similar to what was observed for ibrutinib, acquired resistance to acalabrutinib also resulted in overexpression of these miRNAs in CLL and DLBCL cells and downregulation of PTEN, revealing aberrant expression of 14q32 cluster miRNAs as a common mechanism of resistance to BTK inhibition. Overexpression of the 14q32 cluster miRNAs has been associated with the CCCTC-binding factor (CTCF)-mediated regulation of the maternally expressed gene 3 differentially methylated region (MEG3-DMR) [31] or global genomic hypomethylation of 14q32 locus as reported in various cancers [32], including CLL [33]. While methylation has been well documented in GC-DLBCL [34] there are limited reports on the role of methylation in the ABC-subtype of DLBCL that we have examined as this subtype is responsive to BTK inhibitor therapy. However, chronic exposure to BTKi that induces global hypomethylation of the 14q32 locus that may result in the upregulation of 14q32 miRNAs cluster in BTKi-resistant in comparison to parental CLL and ABC-DLBCL cells warrants further investigation. Importantly, we show increased expression of miR-494, miR-495, and miR-543 and PTEN downregulation in response to BTK inhibition in therapy-relapsed patient-derived primary CLL cells vs those treatment-naïve. Therefore, these clinically relevant data along with our BTKi-R models support a broader mechanism of therapeutic resistance that may be critical for conferring resistance to BTK inhibition.

miRDB target prediction software identified six miRNAs that target PTEN, among which miR-494 had the highest prediction score (Table 1). A target with a prediction score >80 is associated with a high confidence level of the validity of the findings (miRDB.org) [26, 27]. We focused on miR-494 for two reasons: (i) miRDB analysis identified two conserved complementary 8-mer sequences in the 3′-UTR of *PTEN* mRNA that miR-494 is likely to base-pair with, (ii) its location at 14q32, the aberrantly regulated chromosome 14 region that has been previously described in B-cell lymphomas [23]. In support of our preclinical findings, other studies also reported the involvement of 14q32 cluster cancer-related miRNAs in promoting chemotherapy resistance and malignant transformation in various carcinomas [30, 35–44].

**Table 1:** Putative binding sites of 14q32 cluster miRNAs to PTEN 3′ UTR.

miRNA	Target score	Target rank	Conserved sites* (Nr/ position)	Non-conserved sites** (Nr/ position)	Site type
miR-494	97	18	2 (2313, 2798)	3 (5217, 5861, 5905)	8-mer
miR-495	90	60	2 (3221, 3232)	2 (2153, 6272)	8-mer
miR-543	69	160	1 (2311)	2 (3929, 6216)	8-mer
miR-337	92	48	-	1 (109)	8-mer
miR-889	80	100	-	4 (1290, 1616, 2176, 6260)	8-mer
miR-433	67	308	-	3 (3743, 5009, 5128)	7–8-mer

*Conserved sites as shown in Fig. 4f.

**Non-conserved/poorly conserved sites are shown as dotted lines in Fig. 4f.

We provide evidence that BTK inhibition downregulates 14q32 cluster miRNAs and upregulates PTEN in CLL and DLBCL cell lines, an observation that is strengthened by our findings in patient-derived primary CLL cells treated with BTK inhibitors in vitro (Fig. 3c, d) and in samples from patients undergoing ibrutinib therapy in the clinic.

Importantly, we define PTEN as a direct target of miR-494 in CLL and DLBCL cells by two independent approaches: (i) miR-494 modulation both by an inhibitor and a mimic, and (ii) a luciferase reporter assay, consistent with independent investigations showing PTEN as a direct target in various cancers, including hepatocellular carcinoma [42], colorectal cancer [45], and non-small cell lung cancer [46].

We identified 14q32 cluster region miRNAs, such as miR-494, miR-495, and miR-543, to be associated with BTKi resistance and demonstrated that miR-494 mediated PTEN downregulation and AKT activation was responsible for decreased apoptosis. Consistently, our previous studies demonstrated that inhibition of PI3K/AKT signaling sensitizes IB-R cells to apoptosis in a PTEN- and BIM-dependent manner [18]. Now we show that miR-494-mediated PTEN regulation is involved more broadly in BTK-resistance through AKT activation. Pharmacologic AKT inhibition potentiates miR-494 or miR-495 inhibition-induced apoptosis in BTKi-R CLL and DLBCL cells.

**Fig. 7:**
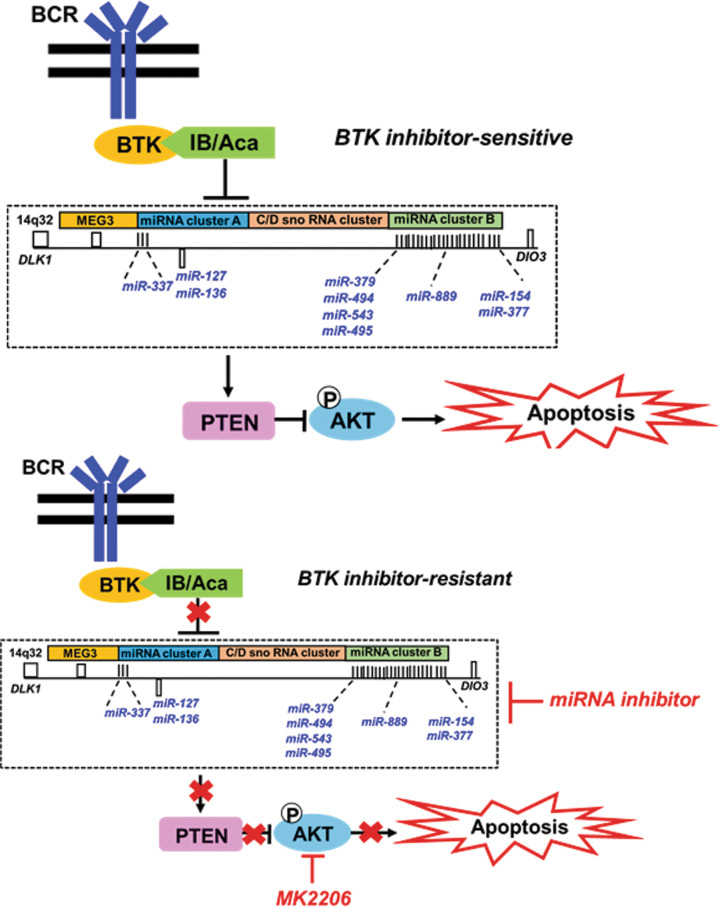
Schematic representation of 14q32 cluster miRNAs-dependent resistance to BTK inhibition in B-cell lymphoid malignancies. BTK inhibitor (ibrutinib or acalabrutinib) downregulates miRNAs in the 14q32 cluster region, such as miR-494, miR-495, miR-543, resulting in increased PTEN expression and induction of apoptosis via antagonizing AKT in BTKi-sensitive cells (upper panel). In BTKi-R cells, overexpressed miRNAs in the 14q32 cluster region downregulate PTEN and promote pro-survival AKT activation resulting in reduced apoptosis. AKT inhibition or cooperative miRNA inhibition rescues apoptosis in BTKi-R cells by restoring PTEN and inhibition of AKT.

Several studies have reported that tumor-promoting miRNAs targeting *PTEN*, such as miR-494 are involved in drug resistance [42, 46], and that their inhibition by anti-miRNA-based therapeutic strategies induce sensitization to apoptosis [42]. Consistently, we show that inhibition of miR-494 or miR-495 either alone or in combination potentiates induction of apoptosis in BTKi-R cells. Moreover, previously, we and others have shown coordinated therapeutic regulation of miRNAs from the 14q32 cluster region, spanning from the *DLK1* to *DIO3* genes, also known as the *DLK1-DIO3* region [23, 28, 32, 47, 48]. Several of these miRNAs, miR-494 [40, 45], miR-495 [49], miR-543 [38, 44], but also miR-889 [38], miR-337 [41], and miR-433 [41] are all targeting the PTEN 3′-UTR (Fig. 7). Importantly, simultaneous inhibition of two miRNAs located in the 14q32 cluster region potentiates the inhibitory action of the anti-miRNA-based strategy and coordinately sensitize BTKi-resistant cells to apoptosis.

AKT/mTOR signaling activation has been characterized as an important resistance mechanism in IB-R mantle cell lymphoma [50], Waldenstrom macroglobulinemia [51], CLL, and DLBCL [18], as well as venetoclax-resistant [52], or fludarabine-resistant [53] B-cell lymphoid malignancies, where inhibition represented a powerful approach to overcome drug resistance and induce apoptosis. Consistently, activation of AKT and downstream targets of mTOR signaling was elevated in our BTKi-R cells, thus making BTKi-R cells more sensitive to AKT inhibition and induction of apoptosis. Indeed, miR-494 or miR-495-dependent inhibition diminished AKT/mTOR activation and sensitized cells to apoptosis. These findings suggest the therapeutic ability of an anti-miRNA-based strategy to block adaptive signaling responses in resistant subclones to overcome drug resistance and induce apoptosis. Previous studies of AKT inhibition in lymphoma patients with MK2206 have shown modest clinical activity [54]. However, recently, large phase III trials in advanced prostate and breast cancer have shown significant improvement in progression-free survival by the addition of the AKT inhibitors ipatasertib [55] and capivasertib [56] to standard treatments. Due to the potential for resistance to continuous treatment with BTK inhibitors, the addition of an AKT inhibitor to such treatment regimens is a rationale strategy.

In summary, our findings provide novel molecular insights into BTK inhibitor resistance mechanisms beyond point mutations in BTK or PLC-γ and support a link between aberrant expression of the 14q32 cluster miRNAs in Aca-R and IB-R cells and the ability of anti-miR-494 or miR-495 to upregulate PTEN to overcome drug resistance and induce apoptosis by diminishing AKT activation (Fig. 7). Importantly, cooperative inhibition of miRNAs leading to induction of apoptosis exploits the resistant cells’ dependency on PTEN/AKT via coordinate regulation of multiple PTEN-targeting miRNAs residing in the 14q32 cluster. Thus, the 14q32 miRNAs cluster/PTEN/AKT/mTOR axis emerges as a determinant of acquired BTKi-R in CLL and DLBCL. Overexpression of miR-494 or miR-495 downregulates PTEN and promotes pro-survival AKT activation in acquired BTKi resistance. Therefore, we propose a combination of miRNA and AKT inhibition as a rational combination strategy to sensitize BTKi-R cells to apoptosis.

## Materials and methods

### Cell lines and patient samples

Human cell lines MEC-1 (CLL) and ABC-DLBCL (RIVA and TMD8) were cultured in RPMI-1640 medium supplemented with 10% FBS (Atlanta Biologicals, Lawrenceville, GA), and antibiotic-antimycotic (Gibco, Life Technologies, Gaithersburg, MD). Ibrutinib-R (IB-R) and Acalabrutinib-R (Aca-R) cells were cultured with 5% FBS. Cell lines were routinely screened for *Mycoplasma*, variations in growth rates, changes in morphological characteristics, and their response to stress with Annexin V FITC-PI staining; their passage number did not exceed 20. Ibrutinib was obtained from Santa Cruz (Chicago, IL); Acalabrutinib (ACP-196) from Selleck Chemicals (Houston, TX).

Peripheral blood samples were obtained from CLL patients after informed consent according to protocols approved by the institutional review board (IRB) according to the Declaration of Helsinki. Patient characteristics were as described earlier [18]. Lymphocytes from these blood samples were purified by Ficoll-Paque PLUS (Amersham Biosciences, Piscataway, NJ) gradient centrifugation.

### Generation of acalabrutinib-resistant (Aca-R) cell lines

Acalabrutinib-resistant TMD8 cells were generated by in vitro culture of the parental cell lines for prolonged periods of time with progressively increasing concentrations of acalabrutinib. Briefly, cells were intermittently incubated with a low concentration (six-fold lower than IC_50_) of acalabrutinib for short intervals over time and allowed to recover after washing off the drug. The acalabrutinib concentration and treatment time were gradually increased until cells remained viable after continuous exposure to the drug that was double the concentration of their IC_50_ value. The Aca-R cells were routinely tested for resistance to acalabrutinib and cultured without the drug for 72 h before they were used in experiments, as described previously for IB-R cells [18].

### Cell viability and apoptosis assays

The number of viable cells in culture was determined by 3-(4,5-dimethylthiazol-2-yl)-5-(3-carboxymethoxyphenyl)-2-(4-sulfophenyl)-2H tetrazolium inner salt (MTS) assay (Promega, Madison, WI), and the percentage reduction in metabolic activity was calculated as previously described [18].

The percentage of cells undergoing apoptosis was measured by phosphatidylserine externalization using fluorescein-conjugated Annexin V/PI double staining (BD Biosciences, San Jose, CA). The analysis was done on a BD FACS MACSQuant flow cytometer (BD Biosciences), and the raw data were processed using the FlowJo software. The results were normalized to the survival of control cells that have been treated with DMSO.

### Immunoblotting

To prepare lysates, cells were collected and washed twice with ice-cold PBS. Cell lysates were prepared in RIPA buffer (20 mM Tris (pH 7.5), 150 mM NaCl, 1 mM EDTA, 1 mM EGTA, 1% Triton X-100, 1% phosphatase inhibitor cocktail (Sigma, St. Louis, MO), and 1 mM PMSF (Sigma) for 30–45 min at 4 °C. The protein concentration in each sample was determined using the Bradford reagent (Bio-Rad, Hercules, CA); 50 µg protein was resolved on 10% SDS-PAGE followed by transferring to nitrocellulose membrane (Millipore, Danvers, MA). The immunoblotting was performed with primary antibodies for PTEN Cat No. #9188 S, cleaved caspase 3 Cat No. #9661 S, AKT Cat No. #9272, pAKT^Ser473^ Cat No. #9271 S, pP70S6^T389^ Cat No. #9205 S, pP70S6 Cat No. #2708 S, p4EBP1^Ser65^ Cat No. #9451 S, pBTK^Y223^ Cat No. #5082 S BTK Cat No. #8547 S (Cell Signaling Technologies, Danvers, MA), GAPDH Cat No. sc-365062 (Santa Cruz Biotechnology, Santa Cruz, CA). The secondary HRP-conjugated anti-mouse Cat No. 31450 and -rabbit Cat No. 31460 antibodies were purchased from Thermo Fisher Scientific (Pittsburgh, PA). The immunoreactive bands were visualized by chemiluminescence according to the manufacturer’s recommendations (Thermo Fisher, Waltham, MA).

### Luciferase reporter assay and miRNA modification

Wild-type and mutant PTEN 3′-UTR luciferase reporters were kind gifts from Dr. Chuanshu Huang (NYU School of Medicine, Tuxedo, NY) [25]. Luciferase assays were performed as described previously [23]. Briefly, TMD8 and TMD8-IB-R cells (30,000 cells/well) were placed in a 24-well plate and 24 h later were co-transfected using Lipofectamine 3000 (Invitrogen-Thermo Fisher, Waltham, MA), with 100 ng WT-3′-UTR or Mut-3′-UTR luciferase reporter constructs, 0.5 ng renilla luciferase reporter plasmid (Promega, Madison, WI) and either miR-494/495-inhibitor (50 nM), -mimic (50 nM), or negative control. Cell lysates were assayed for firefly and renilla luciferase activities 48 h after transfection using the dual-luciferase reporter assay system (Promega, Madison, WI) and a Victor [3] multilabel plate reader (Perkin Elmer, Waltham, MA). Renilla luciferase activity served as a control for transfection efficiency. Data were shown as the ratio of firefly luciferase activity to renilla luciferase activity.

### RNA isolation and real-time quantitative-PCR

Total RNA was extracted using the TRIZOL reagent (Life Technologies) from parental and BTKi- R cell lines or CLL primary cells after ibrutinib or acalabrutinib treatment according to the manufacturer’s instructions. One microgram of the RNA samples was reverse-transcribed using the TaqMan reverse transcription kit and amplified using the SYBR Green Master Mix (Applied Biosystems) and examined on a 7500 Real-Time PCR system (Applied Biosystems, Waltham, MA). Levels of mRNA were analyzed using a quantitative real-time reverse transcriptase PCR (qRT-PCR) kit with primers synthesized by IDT^R^ for *pten* (forward: 5′-CCAATGTTCAGTGGCGGAACT-3′; reverse: 5′-GAACTTGTCTTCCCGTCGTGTG-3′), as described previously [18]. The intensities of each band were normalized to the corresponding *β-actin* bands.

For miRNA analyses, Megaplex RT primers (Applied Biosystems, Waltham, MA), which are 380 stem-looped reverse transcripts that allow cDNA synthesis for mature miRNAs were used except miRNA cDNA synthesis was performed using primer 5′-CAGTGCGTGTCGTGGAGT-3′. The TaqMan MicroRNA Reverse Transcription Kit (Applied Biosystems) was used to make cDNAs for mature miRNAs. The SYBR Green Master Mix (Applied Biosystems) was used to amplify miR-494 using specific primers (forward: 5′-GGGTGAAACATACACGGGA-3′; reverse: 5′- GTCGTATCCAGTGCGTGTCGTGGAGTCGGCAATTGCACTGGATACGACGAGGTT-3′), miR-495 (forward: 5′-GCCAAACAAACATGGTGCACTT-3′; reverse:5′-GTTGGCTCTGGTGCAGGGTCCGA GGTATTCGCACCAGAGCCAACAAGAAG-3′); miR-377 (forward: 5′-GAGCAGAGGTTGCCCTTG-3′; reverse: 5′-ACAAAAGTTGCCTTTGTGTGA-3′); miR-154 (forward:5′-TAGGTTATCCGTGTTGCCTT-3′; reverse: 5′-AATAGGTCAACCGTGTATGATTC-3′); miR-136 (forward: 5′-GGACTCCATTTGTTTTGATGATG-3′; reverse: 5′-AGACTCATTTGAGACGATGATGG-3′); miR379 (forward: 5′-AGAGATGGTAGACTATGGAACGT-3′; reverse: 5′-GTGGACCATGTTACATAGGTCAG-3′), miR-127 (forward: 5′-AGCCTGCTGAAGCTCAGAGG-3′; reverse: 5′-GCCAAGCTCAGACGGATCC-3′), miR-337 (forward: 5′-ACACTCCAGCTGGGTCAAGAGCAAT-3′; reverse: 5′-CTCAACTGGTGTCGTGGA-3′), miR-543 (forward: 5′CCAGCTACACTGGGCAGCA GCAATTCATGTTT-3′; reverse: 5′-CTCAACTGGTGTCGTGGA-3′). The U6 small nuclear RNA primers (forward: 5′- CTCGCTTCGGCAGCACA-3′; reverse: 5′-AACGCTTCACGAATTTGCGT-3′) was used as an internal normalization control since the levels did not change in primary CLL patients’ samples and CLL or DLBCL cell lines.

miRNA overexpression or knockdown was achieved using a specific miRNA mimic or inhibitor or miControl (Ambion, Life Technologies, Austin, TX) by the AMAXA Nucleofector Kit V (Lonza, Walkersville, MD) according to the manufacturer’s protocol.

### Statistical analysis

Each experiment was repeated at least three times. For all the quantitative analyses represented in the graphs, the values are expressed as the mean values ± SD. The significance of the differences between mean values were assessed using a two-tailed Student’s *t*-test and a one-way ANOVA with Bonferroni’s multiple comparison test was performed. All comparisons were calculated using Microsoft Excel version 2106 and GraphPad Prism version 5.00.

## Supplementary information

Supplemental Figure Legends

Supplemental Figure S1

Supplemental Figure S2

Supplemental Figure S3

Supplemental Figure S4

Supplemental Figure S5

## Data Availability

All data generated or analyzed during this study are included in this published article [and its supplementary information files].
