# Supplementary material for: Cooperative miRNA-dependent PTEN regulation drives resistance to BTK inhibition in B-cell lymphoid malignancies
Source: Cell Death Dis. 2021 Nov 8;12(11):1061. doi: 10.1038/s41419-021-04353-9 (PMC8575967; doi:10.1038/s41419-021-04353-9)
Supplement: Supplementary file 1 — Supplemental Figure Legends [file 41419_2021_4353_MOESM1_ESM.doc]

**Supplementary Figure Legends**

**Supplementary Fig. S1 BTK inhibitor treatment downregulates miRNAs in the 14q32 cluster region in CLL and DLBCL cells a** TMD8 cells were treated with the indicated concentrations of acalabrutinib for 72 h and cell viability was determined by the MTS assay. Control cells were treated with DMSO. All data are expressed as mean + S.D. of percentage of cell death. Standard deviation (SD) is indicated as error bars (*N=3*).

**Supplementary Fig. S2 PTEN is a direct target of miR-494 in CLL and DLBCL a** Expression levels of PTEN mRNA and protein in MEC-1-IB-R and **b** RIVA-IB-R cells, respectively after transfection with miR-494 inhibitor or negative control, as indicated. GAPDH was used as a loading control. **c** Expression levels of PTEN mRNA and protein in MEC-1 and **d** RIVA cells, respectively after transfection with miR-494 mimic or negative control, as indicated. GAPDH was used as a loading control. SD is indicated as error bars (*N=3*). *(**p<0.01, ***p<0.001*).

**Supplementary Fig. S3 BIM is a direct target of miR-494 in CLL and DLBCL a** Expression levels of *bim* mRNA in TMD8-Aca-IB-R, **b** TMD8-IB-R, and **c** MEC-1-IB-R cells, respectively after transfection with miR-494 inhibitor or negative control, as indicated. **d** Expression levels of *bim* mRNA in MEC-1 and **e** TMD8 cells, respectively after transfection with miR-494 mimic or negative control, as indicated. The U6 small nuclear RNA was used as internal normalization control. SD is indicated as error bars (*N=3*). *(**p<0.01*). **f** TMD8 and **g** TMD8-Aca-R cells were transfected with miR-494 mimic and miR-494 inhibitor, respectively together with miRControl and ibrutinib (IB) or acalabrutinib (Aca), respectively for 24 h. Cell viability was determined by Annexin V-PI staining. Control cells were treated with DMSO. *(***p<0.001*). All data are expressed as mean + S.D. of percentage of cell death. SD is indicated as error bars (*N=3*).

**Supplementary Fig. S4 miRNA inhibition potentiates AKT-induced apoptosis in BTK inhibitor-resistant CLL and DLBCL a** MEC-1-IB-R cells were transfected with miR-494 and **b** miR-495 inhibitors together with miRControl and treated with MK2206 (5 µM) for 24 h. Cell viability was determined by Annexin V-PI staining. Control cells were treated with DMSO. *(**p<0.01, ***p<0.001*). All data are expressed as mean + S.D. of percentage of cell death. SD is indicated as error bars (*N=3*).

**Supplementary Fig. S5 miR-494 and miR-495 inhibition regulates the AKT/mTOR pathway and enhances cell survival in BTKi-resistant CLL and DLBCL cells a (**Upper panel) MEC-1-IB-R cells were transfected with miR-494 (200 nM) and **b** miR-495 inhibitors (100 nM) and treated with +/- ibrutinib (10 µM) for 24 h. Cell death analysis was determined by Annexin V-PI staining. Control cells were treated with DMSO. *(**p<0.01*). SD is indicated as error bars (*N=3*). (Lower panel) Expression levels of PTEN, pAKT Ser473, AKT, p-P70S6-T389, p-P70S6, p-4EBP1 Ser65, and cleaved caspase 3 were determined in whole-cell extracts of MEC-1-IB-R cells transfected with miR-494 (200 nM) and miR-495 (100 nM) inhibitors, respectively by immunoblotting. GAPDH was used as a loading control.
