# Supplementary figures and images for: Cooperative miRNA-dependent PTEN regulation drives resistance to BTK inhibition in B-cell lymphoid malignancies
Source: Cell Death Dis. 2021 Nov 8;12(11):1061. doi: 10.1038/s41419-021-04353-9 (PMC8575967; doi:10.1038/s41419-021-04353-9)

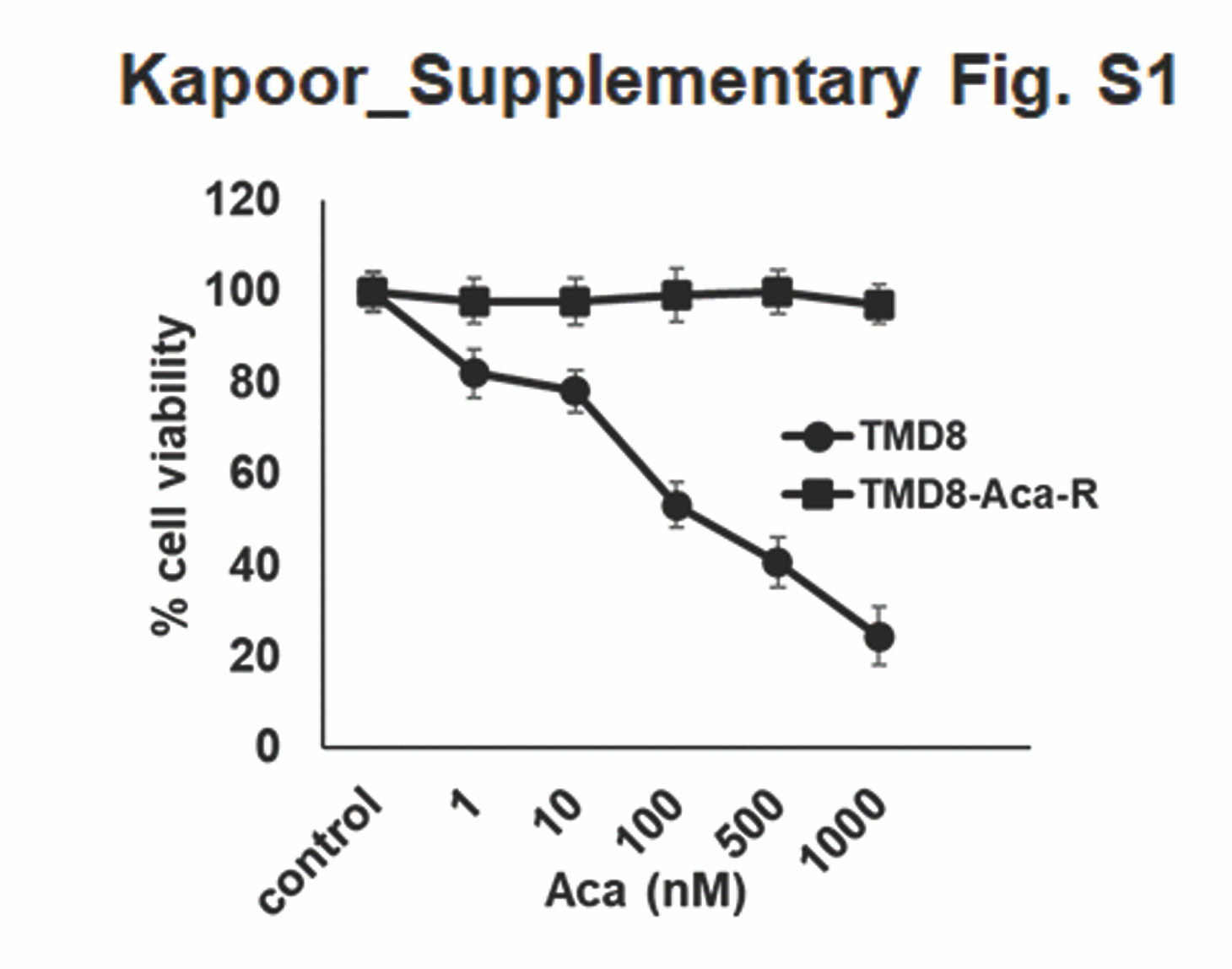

Supplement: Supplementary file 2 — Supplemental Figure S1 [file 41419_2021_4353_MOESM2_ESM.tif]

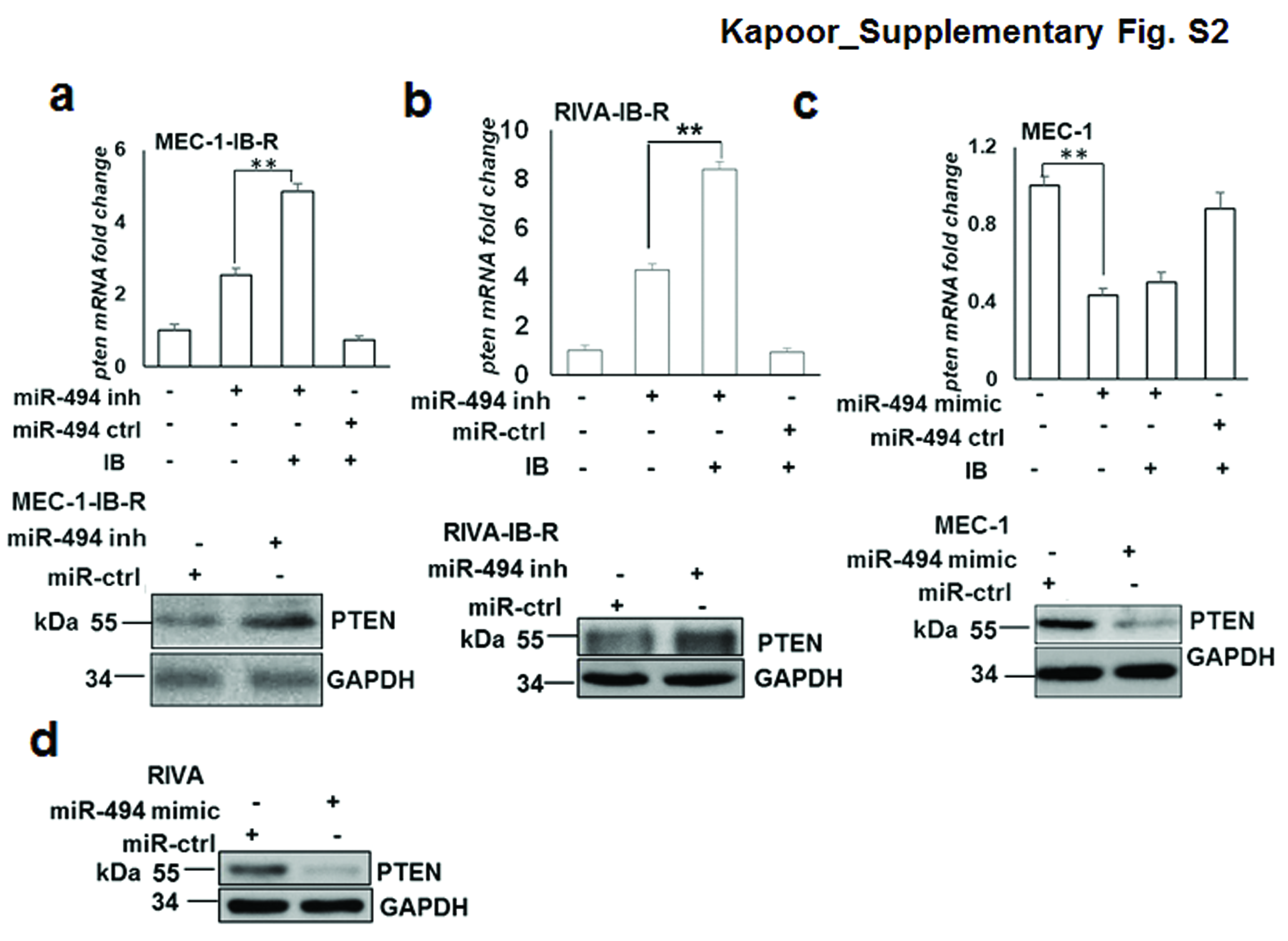

Supplement: Supplementary file 3 — Supplemental Figure S2 [file 41419_2021_4353_MOESM3_ESM.tif]

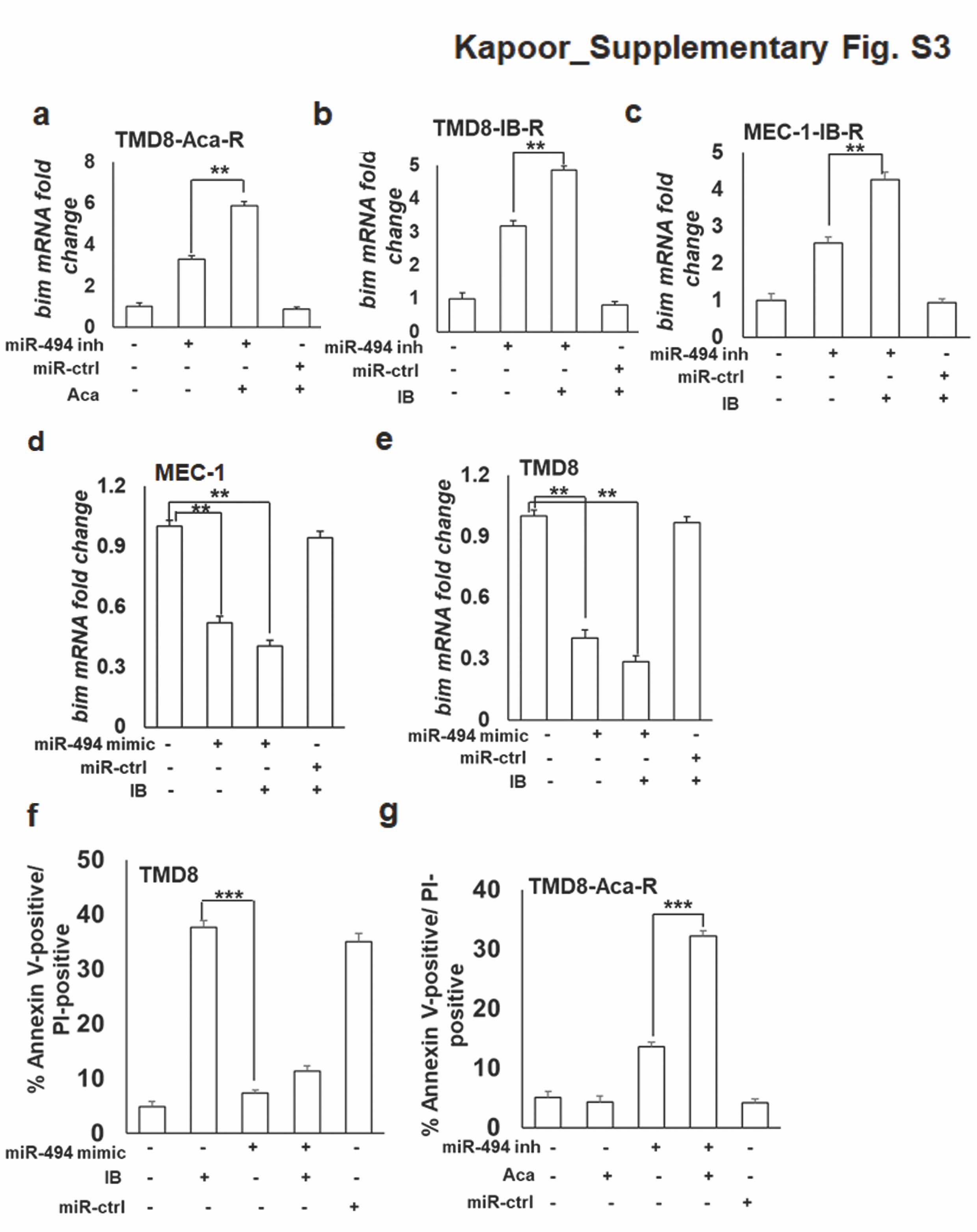

Supplement: Supplementary file 4 — Supplemental Figure S3 [file 41419_2021_4353_MOESM4_ESM.tif]

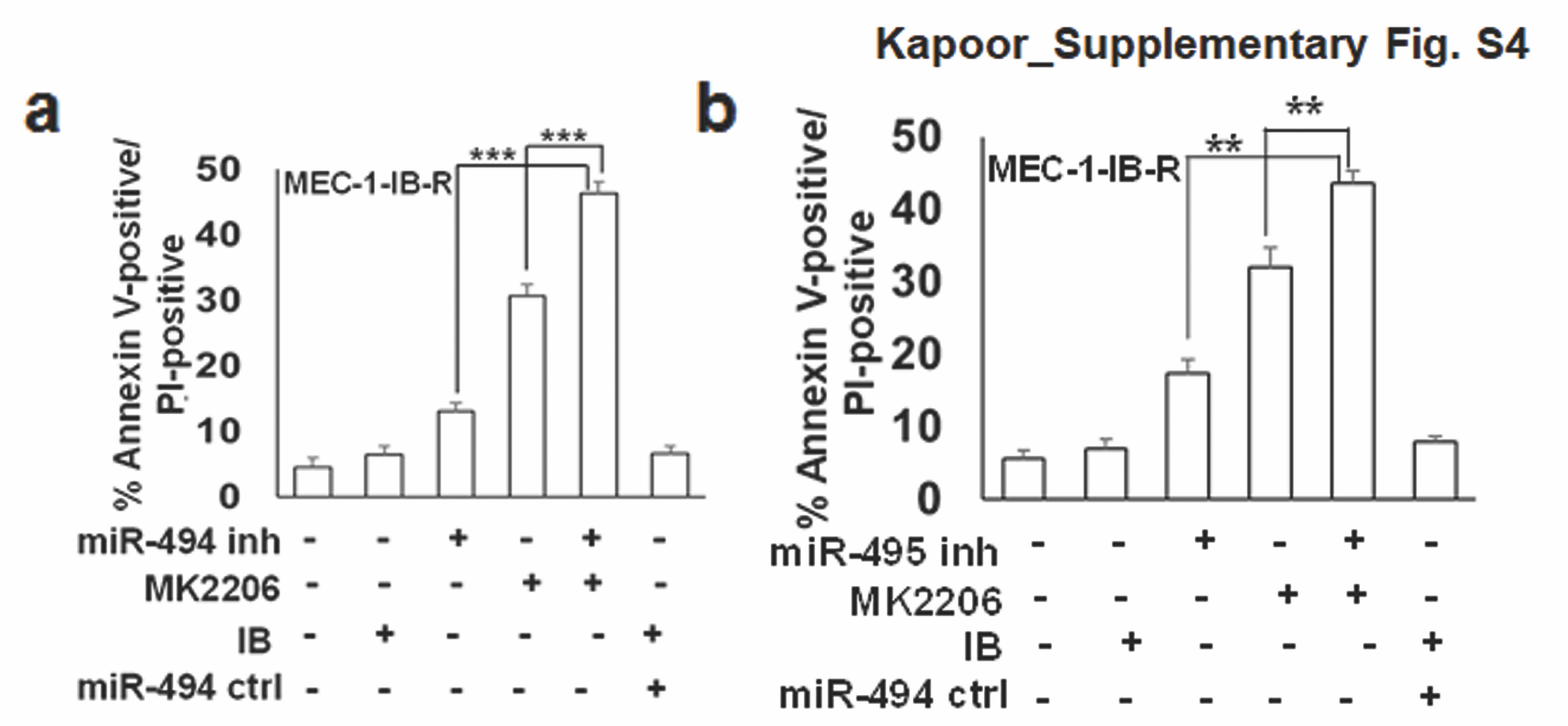

Supplement: Supplementary file 5 — Supplemental Figure S4 [file 41419_2021_4353_MOESM5_ESM.tif]

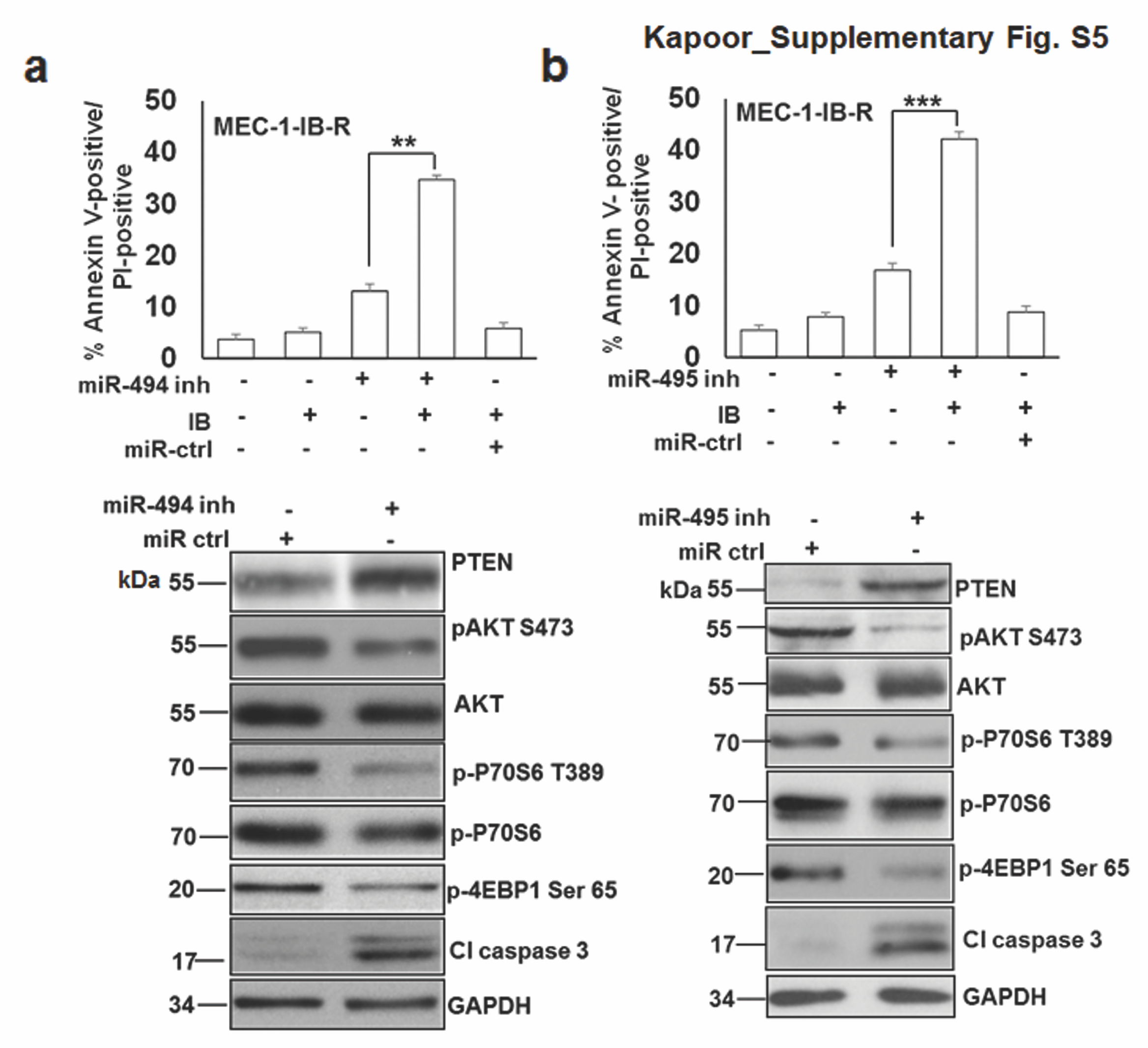

Supplement: Supplementary file 6 — Supplemental Figure S5 [file 41419_2021_4353_MOESM6_ESM.tif]
